# Editorial: Microbial Response to a Rapidly Changing Marine Environment: Global Warming and Ocean Acidification, Volume II

**DOI:** 10.3389/fmicb.2022.1094511

**Published:** 2022-12-01

**Authors:** Mi Sun Yun, Jun Sun, Connie Lovejoy, Sang Heon Lee

**Affiliations:** ^1^Institute for Advanced Marine Research, China University of Geosciences, Guangzhou, China; ^2^College of Marine Science and Technology, China University of Geosciences, Wuhan, China; ^3^Département de Biologie, Université Laval, Quebec, QC, Canada; ^4^Department of Oceanography, Pusan National University, Busan, South Korea

**Keywords:** microbial community, marine environment, ecosystem, warming, ocean acidification

## Introduction

Warming and acidification are representative of ongoing pronounced changes in the world's oceans today. Increasing sea water temperature adjusts basal metabolic rates or physiological status of marine organisms (Reid et al., 2019), and potentially forces some species to shift their distribution ranges (Benedetti et al., 2021). Ocean acidification results in physiological stress of organisms, inhibits their growth, and decreases biological calcification rates, although the degree and direction of these effects vary among taxonomic groups. For this Research Topic, we have focused on the responses of microbial communities. As a vital component of the marine ecosystem microbes play pivotal roles, not only in pathways of energy transfer through the food web but also in global biogeochemical cycles (e.g., Falkowski and Raven, 2013). This Research Topic was conceived to contribute to the understanding of present and future changes in microbial communities in recognition of ongoing warming and acidifying oceanic conditions.

The first volume of this Research Topic on *Microbial response to a rapidly changing marine environment: Global warming and ocean acidification* was launched in 2020 with a total of 10 articles published, covering the wide scope of physiological and ecological responses of diverse taxonomic groups to environmental changes in a range of geographic regions, as summarized in our Editorial (Yun et al., 2021). Due to the success of the first volume, we launched volume II of the Research Topic in 2021. We now add a total of 11 new fascinating articles of which many expand our knowledge on specific aspects of physiological responses to environmental changes. Several articles focused on the alterations of dissolved organic matter (DOM) by bacteria and algae under warming and acidifying conditions, and other works used ecological and model based approaches to examine spatio-temporal dynamics.

## Overview of manuscripts in this Research Topic

Out of the 11 new contributions published, six are focused on the physiological or metabolic response of various microbial groups through field observations or laboratory trials. Billaud et al. demonstrated the effect of elevated seawater temperature on the expression of genes implicated in adhesion and biofilm formation on abiotic surfaces in a strain of *Vibrio parahaemolyticus*. The authors showed that increasing temperature triggers a rapid and transient expression of genes coding for adhesion to plastic surfaces and biofilm development, especially for free living cells. Liang et al. described the physiological and biochemical changes in *Nannochloropsis oceanica* in response to short- and long-term acidification conditions. They found that *N. oceanica* used specific mechanisms to adapt to acidification by regulating carbon and nitrogen metabolism, or changing cellular metabolic components (e.g., fatty acid synthesis). The composition, concentration, and production rates of phytoplankton pigments in the warming and oligotrophic Philippine Sea were investigated by Lee et al. They found that photosynthetic pigments had a significantly faster turnover rate at the surface to harness light energy to repair PSII subunits damaged by strong light. Their findings emphasized the importance of light conditions on phytoplankton physiology in warming ocean scenarios. The responses of phytoplankton photophysiology to nitrogen availability and light conditions at the Subsurface Chlorophyll Maximum (SCM) in the Arctic Ocean were described by Ko et al. The results showed a decrease in nutrient availability in the SCM reduces the photosynthetic activity and large size fraction of phytoplankton. They anticipated that alterations in nutrient flux and light conditions in the SCM in the future Arctic Ocean would be important for regulating phytoplankton photosynthesis and primary production. Calbet et al. presented the effect of thermal stress on the growth, ingestion, and respiration rates of three dinoflagellates. They discussed how the response of the different physiological rates to temperature is species- and strain dependent. The importance of distinguishing the effects of long-term adaptation vs. short-term acclimation of marine mixoplankton and protozooplankton to temperature was verified by Calbet and Saiz. They concluded that in protistan grazers, adaptation to temperature confers a selective advantage to warming within a narrow range (i.e., ca. +3°C). Attempts to adapt to much higher temperatures (i.e., +6°C) do not confer any clear physiological advantage within the temporal framework of their experiments (with few exceptions, e.g., the mixotroph *K. armiger*). Their work expanded our knowledge of protist response to predicted increases in ocean surface temperatures.

Other laboratory-based studies, discussed transformations of DOM derived from bacteria and algae under conditions of warming and acidification. For example, Liu et al. investigated the involvement of the bacterium *Bacillus pumilus* in the production and transformation of the DOM derived from cultures of the diatom *Skeletonema dohrnii*. The results showed that under higher temperature and partial pressure of carbon dioxide (pCO_2_) conditions, *S. dohrnii*-derived DOM was dominated by a protein-like signal, which slowly waned over time, becoming increasingly humic-like. Zhang et al. presented data from *Synechococcous* sp., culture experiments, with and without bacteria present, to examine changes in DOM pools at different temperatures. The results showed that warming could enhance the bioavailability of the *Synechococcus* derived DOM, which may be tempered by the involvement of heterotrophic bacteria. The study provided insight into preservation or persistence of the organic carbon pool in the oceans.

Two studies showed details of spatio-temporal dynamics and environmental drivers related to biogeochemical cycles. Kim et al. conducted *in-situ* observations of the temporal dynamics of carbon and nitrogen uptake rates by phytoplankton in Marian Cove, Antarctica. Their results showed that the strong temporal shifts in phytoplankton carbon and nitrogen assimilation were influenced by wind stress. The response of planktonic ciliate communities in the Pacific Arctic Region was presented in the study of Wang et al. The authors suggested that temperature, which is influenced by hydrographic conditions, has a significant role in determining the aloricate and tintinnid ciliate species composition, with Pacific microzooplankton communities moving north with North Pacific waters. Finally, a model was used to assess the impact of ocean acidification on the size-specific growth of diatoms. Zhang and Luo constructed a theoretical model to understand size-specific growth of diatoms under increasing CO_2_ scenarios. The model revealed a unimodal relationship between the simulated growth rate response (GRR) and cell size. The model further revealed that the “optimal” cell size corresponding to peak GRR increased with the magnitude of CO_2_ increase. This metric then diminished with higher cellular carbon demand, leading to a projection of the smallest optimal cell size in the equatorial Pacific upwelling zone. Their study proposed a competitive advantage for middle-sized diatoms, which could be useful in forecasting changes in the diatom community in future acidified high-CO_2_ oceans.

## Conclusion

In recent years, global attention has been turned toward ocean warming and acidification. As a result, studies reporting effects of these environmental perturbations have proliferated, but most of these studies have focused on single marine organisms and fewer on ecosystems (Raven and Beardall, 2021). This Research Topic was aimed to address this imbalance, to showcase how warming and acidification may alter marine microbial ecosystem structure and function. The studies in this Research Topic and similar studies of microbial responses to rapid environmental changes will contribute to understanding general responses of marine ecosystems to global climate change, including warming and acidification. In volume II of this Research Topic, we explored some of the diversity of physiological and metabolic responses of microbes to changing temperature and carbonate chemistry. Taken as a whole, the articles that make up these two volumes have shown that ongoing and future changes lead to profound consequences for microbial communities.

The mechanisms and processes employed by microbial communities and specific microbes to respond to environmental changes or gradients are highly diverse and complex (Dang et al., 2019). Articles in the Research Topic indicated some of the pressing issues and questions related to microbial response to changing ocean conditions. We, the editors, hope that the two volumes of this Research Topic will form the basis of further discussions and will foster future cross-disciplinary research on the effects of global warming and ocean acidification on marine microbes and ecosystems. Finally, we would like to emphasize the importance of long-term surveys, systematic analyses, and modeling to identify biological responses, feedback, and interactions that will continue to improve projections of microbial and ecosystem responses to climate change.

## Author contributions

MY wrote the draft, with input from JS, SL, and CL. The final version was revised by CL. All authors listed have made a substantial, direct, and intellectual contribution to the work and approved it for publication.

## Funding

MY and JS acknowledge funding from the National Key R&D Program of China (2019YFC1407805) and the National Nature Science Foundation of China (41876134). CL acknowledges funding from the Natural Sciences and Engineering Council (NSERC) Canada. Research support for SL was provided by the project titled KIOS (Korea Indian Ocean Study): Korea-US Joint Observation Study of the Indian Ocean, funded by the Korean Ministry of Oceans and Fisheries (20220548, PM63180).

## Conflict of interest

The authors declare that the research was conducted in the absence of any commercial or financial relationships that could be construed as a potential conflict of interest.

## Publisher's note

All claims expressed in this article are solely those of the authors and do not necessarily represent those of their affiliated organizations, or those of the publisher, the editors and the reviewers. Any product that may be evaluated in this article, or claim that may be made by its manufacturer, is not guaranteed or endorsed by the publisher.
